# Epidemiology of COVID-19

**DOI:** 10.3906/sag-2004-172

**Published:** 2020-04-21

**Authors:** Cemal BULUT, Yasuyuki KATO

**Affiliations:** 1 Department of Infectious Diseases & Clinical Microbiology, Gülhane Faculty of Medicine, Health Science University, Ankara Turkey; 2 Department of Infectious Diseases International University of Health and Welfare, Narita Hospital Narita Japan

**Keywords:** SARS CoV-2, COVID-19, epidemiology, case fatality rate, transmission

## Abstract

It seems that coronaviruses take an important place in the 21th century history. Five of seven human coronavirus was isolated in this century. Unfortunately, last three of them entered our life with a fear of outbreak, pandemic or death. Last human coronavirus which emerged world from Wuhan China, SARS CoV-2 and its clinical expression, Coronavirus disease (COVID-19) recently taken a significant place in our daily practice. Initial reports showed that, its origin was bats. It transmitted human to human by droplet and contact routes, but some doubt about airborne, fecal or intrauterine transmission also should be removed. Its R0 value is 2.3 but it could be as high as 5.7. Its case fatality rate was 6.3, but it was different in different ages and counties, and it could be over 15%. According to early models total 10–12 weeks is required to control an outbreak in the community. While different countries show different daily case numbers, total number of case, case mortality rates or R0, it seems they show a similar epidemic curve. Every day we learn new data about the current outbreak. Since the outbreak is not over yet, every detail should be evaluated carefully and the updates should be followed closely to monitor the epidemiological properties of COVID-19.

## 1. Introduction

Coronoviruses have been reported as causes of mild and moderate respiratory infections forover 50 years [1]. Even though this group of viruses have been isolated from many different animals, bats are accepted major natural reservoir of coronaviruses [2,3].

Four human coronavirus, 229E, HKU1, NL63 and OC43, are known as causes of common cold in humans [1]. However, recently-detected coronaviruses, SARS CoV (2002), MERS-CoV (2012) completely altered all known approaches about this virus group because these viruses caused severe acute respiratory infections and nosocomial outbreaks. In the end of 2019, a novel coronavirus, now known as SARS-CoV-2 (2019), suddenly emerged in Wuhan, China. The World Health Organization declared that the epidemic is a public health emergency of international concern on January 31, 2020. As of April 16, 2020, the emerging coronavirus infection, COVID-19, has been spreading worldwide, causing over 2 million cases and over 137 thousand of death.

In as much as this disease recently taken a significant place in our daily practice, as a new group of respiratory diseases, due to their higher rates of transmissibility, hospitalization, intensive care unit admission, severity of disease, mortality and so on, we should pay more attention than past to prevention and treatment of coronavirus infection. 

## 2. Origin of the SARS CoV-2 virus

The first cases of coronavirus disease COVID-19 were directly related to an animal market in Wuhan, China. Early investigations suggested that the origin of SARSCoV-2 may be bats. Zhou et al. demonstrate that SARS CoV-2 possesses 96% nucleotide identity with a bat coronavirus, for instance BetaCoV/RaTG13/2013 [4].

## 3. Epidemic curve of infection

An epidemic curve of infection is a statistical chart used in epidemiology to visualize the onset of the coronavirus outbreak. In an epidemic curve, there are three zones: increasing, plateau, and declining phases. 

**Figure 1:**
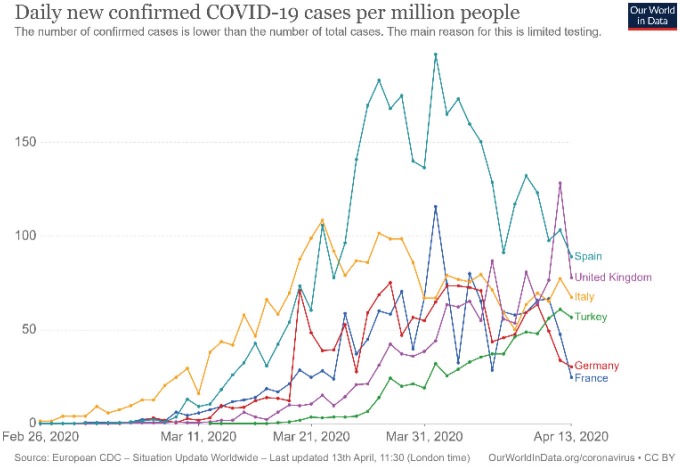
Daily new confirmed COVID-19 cases per million people for some contries (Source: OurWorld in Data, accessed 13 April 2020).

Increasing phase: This period is affected by many different parameters such as country demographics, age distribution, preparedness of health system to an outbreak, implementation of some preventive measures, country reaction time to a pandemic, reaction of society to new implementing rules. Different countries can exhibit quite different curve patterns and these facts could complicate to make any assumption about pandemic pattern for a country (Figure 1). However, it seems that this period takes generally 3 or 4 weeks for COVID-19 (Table 1).

**Table 1:** Dates of epidemic curve (dates for case per million was used)*.

Countries	Onset of outbreak(date)	Onset of plateau(date)	End of plateau phase(date)	End of declining phase(date)	Duration of increasing period (day)	Duration of plateau period (day)	Duration of declining period (day)
China	19.01.2020	05.02.2020	19.02.2020	3.03.2020	17	14	13
Italy	22.02.2020	22.03.2020	08.04.2020	N/A	29	17	N/A
Germany	28.02.2020	20.03.2020	10.04.2020	N/A	21	21	N/A
France	28.02.2020	24.03.2020	11.04.2020	N/A	25	18	N/A
Spain	27.02.2020	26.03.2020	9.04.2020	N/A	28	14	N/A
Turkey	16.03.2020	11.04.2020	N/A	N/A	26		N/A
South Korea	19.02.2020	29.02.2020	10.03.2020	N/A	10	10	N/A
Belgiun	03.03.2020	27.03.2020	12.04.2020	N/A	24	16	N/A
Austria	22.02.2020	20.03.2020	04.04.2020	N/A	27	15	N/A
Australia	05.03.2020	22.03.2020	04.04.2020	N/A	17	13	N/A
Malasia	02.03.2020	16.03.2020	11.04.2020	N/A	14	26	N/A

*According to data’s of 13.04.2020.

Plateau phase: At this phase disease incidence is stable. According to daily country reports it takes 2 or 3 weeks for COVID-19 (Table1).

**Figure 2:**
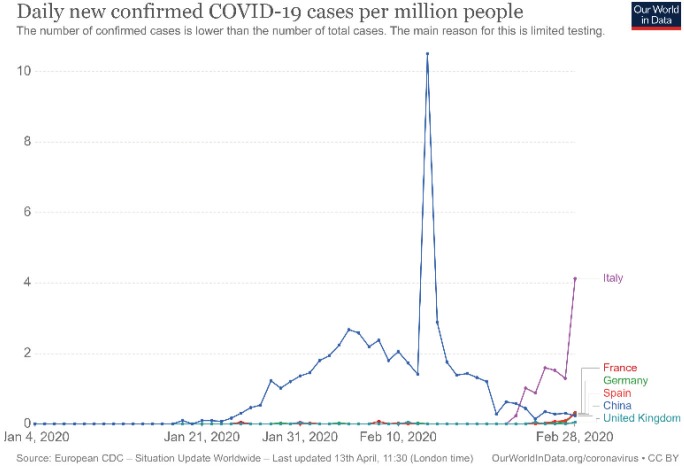
Daily new confirmed COVID-19 cases per million people for China (Source: OurWorld in Data, accessed 13 April 2020).

Decreasing phase: Today we have only China’s data about this phase (Figure 2), showing that 2 or 3 weeks later disease activity could be detected very low levels.

## 4. Incubation period

The incubation period of COVID-19 was defined 5.2 days (95% CI, 4.1–12.5 days), 5.1 days (95% CI, 4.5 to 5.8 days) and 4 days in three separate study [5,6]. However, in a familial cluster of 5 patients, this period was reported between 1 and 19 days [7]. These data show that the incubation period of COVID-19 was similar to MERS and SARS and it’s a bit longer than influenza [8].

## 5. Severity of infection

COVID-19 has been reported form all aged patients. Severity of infection could be varied from asymptomatic infection to critical disease. Clinical severity of COVID-19 was defined in 5 groups as asymptomatic, mild, moderate, severe, and critical. Diagnostic criteria of these groups were as follows [9, 10];

1. Asymptomatic infection: Without any clinical sign of symptoms with positive SARSCoV-2 PCR test.

2. Mild: Symptoms of acute upper respiratory tract infection, including fever, fatigue, myalgia, cough, sore throat, runny nose, and sneezing without pneumonia.

3. Moderate: With pneumonia, frequent fever and cough; some may have wheezing, but no obvious hypoxemia such as shortness of breath.

4. Severe: Rapid progression around 1 week, dyspnea, with central cyanosis, oxygen saturation less than 92%, with other manifestations of hypoxemia.

5. Critical: Patients with acute respiratory distress syndrome (ARDS) or respiratory failure, shock, multiple organ dysfunction.

This clinical classification is also important because it gives some clues about prognosis and mortality of COVID-19. Most COVID-19 cases (81%) were classified as mild or moderate disease in adults, and in children most cases were mild. [9,11,12]. In critical cases mortality could be as higher as 50% in adults [9,11]. 

### 5.1. Asymptomatic infection

Asymptomatic infection has a special importance as a source of disease in the community. Especially, asymptomatic infants and children may take an important role in human-to-human transmission [13]. Dong and colleagues report that over 90% of pediatric patients could be asymptomatic, or could have mild or moderate disease [11]. It seems that asymptomatic infection could be detected at every age, although it was frequent at younger ages [14]. These asymptomatic patients could cause familial clusters [15]. According to Tian et al., asymptomatic infection rate was 5%, whereas in Diamond Princess Cruise Ship berthed at Tokyo Bay, Japan, it was nearly 18% [16,17]. Among a total of 72,314 China’s cases, 889 were classified as asymptomatic (1%) [9]. However, we need more accurate numbers before making some assumption about its public health importance.

## 6. Route of transmission

In spite of the fact that some animals are thought as origins of the virus, a major transmission mode for SARS CoV-2 is human-to-human transmission like SARS CoV and MERS CoV. Accepted transmission routes of COVID-19 are droplet and contact transmission [18]. Nevertheless, explaining or excluding of other possible transmission routes is necessary for controlling disease in the community. 

### 6.1. Airborne transmission

Current evidence shows that SARS CoV-2 was detected in air samples for up to 3 h in experimental models [19]. Gou et al. found that air samples were positive in many different sites of wards [20]. However, when Cheng et al. collected 8 air samples at a distance of 10 cm from the patient’s chin with or without wearing a surgical mask, they were not able to detect SARS CoV-2 [21]. Another study, although some environmental samples were positive for SARS CoV-2, all air samples were negative [22]. In a study from Iran, authors reported that all air samples collected 5 m around the patients were negative [23]. 

There are some limitations in these studies where different patients and methods were included; in which stage of disease these samples were collected, or what kind of procedures were performed. Today, we have not known yet whether detected SARS CoV-2 in air samples are viable or not, or whether detected viral loads are high enough to cause COVID-19. It is important to realize here that prior to answering such questions and obtain some strong evidence, taking airborne precautions for aerosol producing process seems to be logical.

### 6.2. Fecal transmission

Fecal transmission is another unanswered question about COVID-19. Presence of patients with diarrhea [6], finding of fecal samples positive for SARS CoV by RT-PCR [24,25] and detection of live virus in stool at least one study [25], suggest that stool is infectious even though it has not been verified by other studies [24]. Detection of SARS CoV-2 RNA in patients’ fecal samples with negative respiratory tract samples for a mean of 11 days might be important [26]. Even if gastrointestinal involvement or digested sputum was the origin of virus existence in the stool, this fact gives another reason to emphasize good personal hygiene. Possibility of fecal transmission remains unanswered until viable virus is detected in stool samples and new data is available to prove oral-fecal transmission, which affect procedures for quarantine, hospitalization and self-isolation practices.

### 6.3. Maternal fetal transmission

Intrauterine or transplacental transmission from infected pregnant women to their fetuses is another important point for transmission. According to a study about analyzing of 38 pregnant women with COVID-19, there were no signs of intrauterine transmission of SARS CoV-2 from mothers to their fetuses [27]. Studies also emphasized that the course of COVID-19 was normal in pregnant women as opposed to those of SARS and MERS [27,28]. Another study also reviewed that we have no definitive evidence of vertical transmission [29].

## 7. Transmission period

People infected with SARS CoV-2 can be contagious prior to onset of symptoms. In a study, 13% of patients were contagious before onset of symptoms [30]. In a familial cluster, 3 of 8 laboratory-confirmed patients were asymptomatic [31]. Presymptomatic transmission would be a challenge to contact tracing [32]. These kind of reports show that asymptomatic carriers emerge as an important target group to be taken into consideration in the control of the disease.

Viral shedding might be longer than previously thought. In a retrospective multicenter cohort study of 191 adult patients, the median duration of viral shedding was found to be 20 days (IQR 17.0–24.0) and the longest observed duration of viral shedding in survivors was 37 days [33].A study of postdischarge surveillance of patients after clinical recovery, two patients were found positive [34].

## 8. Susceptible ages

Although COVID-19 was reported from every aged patient, patients with advanced age seem more susceptible to infection. An early report shows that the most affected age group was middle ages (Table 2). Among children and young adults, infection rates were very low and it changed between 0.8%–4.0%. In addition, asymptomatic infection rate is quite high in this ages. 

**Table 2:** Confirmed COVID-19 cases by age groups.

	Spain*		Canada**		Nederland***		China****		Italy*****		Germany^Y^	
	Total case	%	Total case	%	Total case	%	Total case	%	Total case	%	Total case	%
<20	741	0.8	220	4	173	1.1	965	2	1868	1.5	2624	2.8
20–29	4,629	5.2	658	13	974	6.2	3619	8	33,248	26.7	66,896	70.3
30–39	8,761	9.9	847	16	1251	8	38,680	87				
40–49	13,421	15.2	881	17	1582	10.1						
50–59	16,577	18.8	1023	19	2590	16.5			44,581	35.8		
60–69	14,933	16.9	855	16	2450	15.5					18,307	19.3
70–79	14,300	16.2	468	9	3013	19.8			44,830	36		
80–89	14,828	16.8	309	6	3550	22.8	1408	3			7357	7.7
Total	88,190	100.0	5261		15723	100	72,314	100	124,527	100	94,850	100
* Spain (2020), Actualización nº 67. Enfermedad por el coronavirus (COVID-19) [online]. Website https://www.mscbs.gob.es/profesionales/saludPublica/ccayes/alertasActual/nCov-China/documentos/Actualizacion_67_COVID-19.pdf [accessed 06 April 2020].												
** Canada (2020). Age distribution of COVID-19 cases (n=5,261) in Canada [online]. Website https://www.canada.ca/en/public-health/services/diseases/2019-novel-coronavirus-infection/health-professionals/epidemiological-summary-covid-19-cases.html#a3 [accessed 01 April 2020].												
***Nederland (2020). Epidemiologische situatie COVID-19 in Nederland 3 april 2020 [online]. Website https://www.rivm.nl/documenten/epidemiologische-situatie-covid-19-in-nederland-3-april-2020 [accessed 03 April 2020].												
**** China (2020). Characteristics of and important lessons from the coronavirus disease 2019 (COVID-19) outbreak in China [online]. Website https://jamanetwork.com/journals/jama/fullarticle/2762130 [accessed 00 Month Year].												
***** Italy (2020). Integrated surveillance of COVID-19 in Italy [online]. Website https://www.epicentro.iss.it/en/coronavirus/bollettino/Infografica_6aprile%20ENG.pdf [accessed 00 Month Year].												
^Y^Germany (2020). Coronavirus disease 2019 (COVID-19) daily sSituation report of the Robert Koch Institute 06/04/2020-updated status for Germany [online]. Website https://www.rki.de/DE/Content/InfAZ/N/Neuartiges_Coronavirus/Situationsberichte/2020-04-06-en.pdf?__blob=publicationFile [accessed 00 Month Year].												

## 9. Transmissibility and R0

Early reports from China show that a reproductive number for COVID-19 was 2.2 to 2.7 days [5]. This means that the number of infected person will be doubled every 6–7 day. A reproductive number of the outbreak on the Diamond Princess Cruise Ship also supports this preliminary data [35]. However, Sanche et al. found that a serial interval was 5.7 days (95% CI, 3.8–8.9 days) [36]. In another study which prefer to use real-time reproduction number (Rt) instead of basic reproduction number (R0), Yuan et al. showed that their Rt values for Italy, Germany, France and Spain were 3.1, 4.43, 6.56, and 3.95, respectively [37]. Liu et al. found that average R0 was 3.8 (1.4–6.49) in their review of 14 studies [38]. This study also showed that even in the same geographic region, different Ro values can be calculated by using different methods and assumptions. It seems that we need more data to determine more accurate R0 value. 

## 10. Case fatality rate

Overall case fatality rate was 6.3 according to WHO situation report as of April 13, 2020 [39]. Nevertheless, a remarkable difference in mortality rates was also noted between countries as given Table 3. It was noted that this rate is extremely higher in country with older-aged populations. In Italy, the median age of those died from COVID-19 was 78 years, whilst the median age of patients was 62 [40]. In Turkey, the current case fatality rate is 2.1% which is a significant issue to bear in mind. Mortality associated with COVID-19 is a multifactorial process and underlying diseases and health care burden as well as ages could be related to higher mortality [41].

**Table 3:** Total confirmed cases, deaths and case fatality rate of some countries*.

Reporting country	Total confirmed cases	Total deaths	Case fatality rate
France	94,382	14,374	15.23
Italy	156,363	19,901	12.73
The United Kingdom	84,283	10,612	12.59
Belgium	29,647	3600	12.14
Netherlands	25,587	2737	10.70
Spain	166,019	16,972	10.22
Sweden	10,483	899	8.58
İran	71,686	4474	6.24
Brazil	20,727	1124	5.42
China	83,597	3351	4.01
USA	524,514	20,444	3.90
Switzerland	25,220	858	3.40
Portugal	16,585	504	3.04
Canada	23,702	674	2.84
Austria	13,937	350	2.51
Germany	123,016	2799	2.28
Turkey	56,956	1198	2.10
Republic of Korea	10,537	217	2.06
Israel	10,878	103	0.95
Russian Federation	18,328	148	0.81
Grand total	1,773,084	111,652	6.30

*World Healt Organization (2020). Coronavirus disease 2019 (COVID-19) Situation Report–84 [online]. Website https://www.who.int/docs/default-source/coronaviruse/situationreports/ 20200413-sitrep-84-covid-19.pdf?sfvrsn=44f511ab_2 [accessed 13 April 2020].

### 10.1. Mortality by ages

Increasing of mortality with advanced age is now a well-known fact. It is also well known that success in prevention of COVID-19 among these age groups directly determines th emortality rate in countries. Early Chinese reports showed that mortality rate could be 3 times higher in older patients especially those at age over 80 [9]. In an Italian study, ICU mortality was 26% whereas it was 36% after 65 of ages [42]. Demonstration of median days between onset of symptoms to death was shorter in older patients which is another important point [43].

As of April 7, 2020 in Italy, 83% of all COVID-19 related deaths were reported in an age group over 70 years [44]. In a Korean study, although overall mortality rate was 0.9%, mortality rate in those aged 80 and over was 9.3% [45]. Similar results were also reported form USA [45].

## 10.2. Risks for disease and death

Disease progression can be rapid and the median survival time can be as short as 5 days in advanced aged patients [28]. 

In a metaanalysis which evaluated 46,248 patients from eight studies, most prevalent comorbidities were hypertension, diabetes mellitus, cardiovascular diseases and respiratory diseases. Another finding of this study was that these comorbidities were more likely detected in severe patients [47]. Another metaanalysis showed that hypertension, cardiovascular diseases, diabetes mellitus, smoking, chronic obstructive pulmonary disease, malignancy, and chronic kidney disease were most frequently detected underlying diseases among hospitalized patients [48].

In a summary report form China CDC, preexisting comorbid condition can increase fatality rate by 10.5% for cardiovascular disease, 7.3% for diabetes, 6.3% for chronic respiratory disease, 6.0% for hypertension, and 5.6% for cancer [9].

According to the Italian weekly report on death from COVID-19, most frequently detected comorbidities were hypertension, diabetes mellitus, ischemic heart disease, and chronic renal failure as 72%, 31.5%, 27.4%, and 23.5%, respectively. This report showed that patients without any comorbidities accounted for only 2.8% of those died from COVID-19 [40].

In a pooled analysis, risk of severe disease and mortality was higher in hypertensive patients nearly 2.5 fold increases especially among advanced aged individuals [49]. 

## 12. Conclusion

Every day we learn new data about the current pandemic of COVID-19. Since the outbreak has not been over yet, the updates should be followed closely to monitor the disease and the risk factors as well as therapy modalities.

## Acknowledgments/disclaimers

Cemal BULUT, MD, Prof. worked with the Turkish Red Crescent, Ministry of Health and Disaster and Emergency Management Presidency in different disaster such as Pakistan’s Flood Disaster, Somalia, Darfur Sudan, Banda Aceh, Endonesia.

Yasuyuki KATO, MD, MPH, Prof. is the former Chief Physician of Division of Preparedness and Emerging Infections Disease Control and Prevention Center National Center for Global Health and Medicine of Japan.
